# Effects of Remote Immune Activation on Performance in the 5-Choice Serial Reaction Time Task Following Mild Traumatic Brain Injury in Adolescence

**DOI:** 10.3389/fnbeh.2021.659679

**Published:** 2021-04-01

**Authors:** Lola Kaukas, Justin Krieg, Lyndsey Collins-Praino, Frances Corrigan

**Affiliations:** Head Injury Laboratory, Adelaide Medical School, University of Adelaide, Adelaide, SA, Australia

**Keywords:** prefrontal cortex, motivation, attention, traumatic brain injury, neuroinflammation

## Abstract

In adult pre-clinical models, traumatic brain injury (TBI) has been shown to prime microglia, exaggerating the central inflammatory response to an acute immune challenge, worsening depressive-like behavior, and enhancing cognitive deficits. Whether this phenomenon exists following mTBI during adolescence has yet to be explored, with age at injury potentially altering the inflammatory response. Furthermore, to date, studies have predominantly examined hippocampal-dependent learning domains, although pre-frontal cortex-driven functions, including attention, motivation, and impulsivity, are significantly affected by both adolescent TBI and acute inflammatory stimuli. As such, the current study examined the effects of a single acute peripheral dose of LPS (0.33 mg/kg) given in adulthood following mTBI in mid-adolescence in male Sprague–Dawley rats on performance in the 5-choice serial reaction time task (5-CSRTT). Only previously injured animals given LPS showed an increase in omissions and reward collection latency on the 5-CSRTT, with no effect noted in sham animals given LPS. This is suggestive of impaired motivation and a prolonged central inflammatory response to LPS administration in these animals. Indeed, morphological analysis of myeloid cells within the pre-frontal cortex, *via* IBA1 immunohistochemistry, found that injured animals administered LPS had an increase in complexity in IBA1+ve cells, an effect that was seen to a lesser extent in sham animals. These findings suggest that there may be ongoing alterations in the effects of acute inflammatory stimuli that are driven, in part by increased reactivity of microglial cells.

## Introduction

The most common form of traumatic brain injury (TBI) is classified as mild (Bazarian et al., 2006; Langlois et al., 2006), resulting from biomechanical forces causing movement of the brain within the skull, without gross structural pathology (McKee et al., 2015). Clinical symptoms include headache, dizziness, nausea, attention deficits, and difficulty with memory, which typically resolve within 2 weeks, but may persist beyond 4 weeks, in up to 30% of patients (Babcock et al., 2013). Even if symptoms resolve, a prior injury may increase vulnerability to a subsequent inflammatory event, such as infection (Sun et al., 2018) or subsequent mTBIs (McAteer et al., 2016).

A mild TBI damages cells, releasing damage-associated molecular patterns (DAMPs; Relja and Land, 2020) and driving activation of resident microglia and astrocytes, which in turn release pro-inflammatory cytokines and chemokines (Burda et al., 2016; Donat et al., 2017). In a mild injury, levels of these pro-inflammatory cytokines and chemokines increase both within the brain, as well as systemically, returning to baseline typically within 7 days (Shultz et al., 2011), although levels of certain mediators, like CCL2, may remain mildly elevated in some individuals (Sun et al., 2019). Furthermore, microglia themselves may remain primed, leading to an exaggerated response to other inflammatory stimuli and promoting the subsequent development of cognitive deficits (Norden et al., 2015). In support of this, cytokine levels are known to positively correlate with cognitive impairment post-mTBI (Sun et al., 2019). Indeed, in adult animals, a peripheral immune insult at 5 days post-injury led to chronic impairments in cognitive flexibility in the Barnes Maze at 3 months post-injury, an effect not seen with injury alone (Collins-Praino et al., 2018). Even remote inflammatory stimuli can drive this response, with a peripheral immune challenge at 1-month post-injury acutely worsening memory consolidation on the Barnes Maze (Muccigrosso et al., 2016) and enhancing depressive-like behavior on the tail suspension test (Fenn et al., 2014). Cognitive flexibility is strongly associated with medial prefrontal cortical (mPFC) function (Jett et al., 2017), with depressive symptoms linked to decreased mPFC volume related to inflammation (Belleau et al., 2019).

While the effects of TBI on microglial priming have been conducted in adult animals (Fenn et al., 2014; Muccigrosso et al., 2016; Collins-Praino et al., 2018), whether age at the time of injury affects this response has yet to be explored. The immediate inflammatory response to injury differs with age, with pediatric animals showing higher levels of pro-inflammatory markers than young adults (Webster et al., 2019). Investigating whether differences in this acute response in adolescents influence later microglial priming post-injury is of critical importance, given that adolescents have the highest rate of injury (Skandsen et al., 2019) and maybe at increased risk of long-lasting symptoms, occurring in up to 29% of this population, compared to 10–20% of adults. In particular, injury during adolescence may affect executive function (Baillargeon et al., 2012; Howell et al., 2013), which describes higher-order cognitive abilities incorporating attention, initiation of activity, cognitive inhibition, and problem-solving. Indeed, deficits in attention were present up to 2 months following concussion in adolescence (Howell et al., 2013), and adolescents, but not adults, who had suffered a mTBI in the preceding year were found to have deficits in working memory (Baillargeon et al., 2012). Given the known relationship between neuroinflammation and subsequent cognitive impairment, it is reasonable to hypothesize that such executive function deficits may be driven by microglial priming following TBI. To investigate this, we examined whether a remote immune challenge in adulthood exacerbated executive function deficits in animals previously injured during adolescence.

## Materials and Methods

All studies were performed within the guidelines established by the National Health and Medical Research Committee of Australia and were approved by the Animal Ethics Committee of the University of Adelaide. Male Sprague–Dawley rats (from Adelaide University breeding colony) were group-housed in individually ventilated cages in a controlled temperature environment under a 12 h light/dark cycle. Rats were randomly allocated to receive either sham anesthesia or a single mild TBI at postnatal day 35 (p35), which is described as representing mid-adolescence in humans (Spear, 2000). Animals were allowed to recover for 2 weeks, before undertaking training on the five serial choice reaction time task (5-CSRTT). On completion of training, animals were randomly allocated to receive 0.533 mg/kg of LPS (Sigma–Aldrich: *E. coli* 055:B5) or an equal volume of saline (*n* = 12–14 per group) *via* intraperitoneal injection. LPS was received as a lyophilized powder and solubilized in 0.9% saline to a final concentration of 0.33 mg/ml. It was stored at −80°C until shortly before use, where it was stored at 4°C and vortexed for 30 s before drawing up in the syringe. Previous studies utilizing this dose have shown that it produces a low-grade systemic inflammatory response (Wang et al., 2016). Following a rest day for recovery, animals underwent a probe trial on the 5-CSRTT before perfusing fixation with saline (*n* = 7 per group).

### Rodent Model of TBI

As per Mychasiuk et al. (2014), animals were subjected to a closed head weight drop model of diffuse TBI. The model combines a high-velocity impact with rapid head acceleration to mimic clinical concussive injuries (Viano et al., 2007). An mTBI clinically describes a brain injury that leads to transient confusion, with or without loss of consciousness with memory impairment near the time of injury and signs of neurological or neuropsychological dysfunction (Prince and Bruhns, 2017). The model here has previously been described to produce a brief loss of consciousness, seen by an increase in righting reflex and acute impairments in learning and memory on the Morris Water Maze and impaired balance on the ledged beam (Mychasiuk et al., 2014), reflective of an mTBI.

Animals were briefly anesthetized with 3.5% isoflurane in the air for 3 min before being placed chest down on scored tin foil with the head directly in the path of a 100 g weight that was released from a height of 0.75 m. The weight strikes the rat’s head, causing the animal to fall through the foil, undergo a 180° rotation, and land on a foam bed below. When applied to adolescent rats, the diffuse injury model produces behavioral outcomes representative of post-concussive symptoms, including balance difficulties in the acute recovery phase (Prince and Bruhns, 2017). Righting time post-injury was used as confirmation of injury and was measured as the time taken for animals to return to a standing position following removal of anesthetic.

### Five Serial Choice Reaction Time Task (5-CSRTT)

At 19 days post-injury, animals began training on the 5-CSRTT. Behavioral testing was conducted in the Bussey–Saksida Touchscreen operant chamber (Campden Instruments Limited, UK) as previously described (Collins-Praino et al., 2018; Kaukas et al., 2021). Animals were food-restricted for 5 days before starting training (at 14 days post-injury), receiving 5 g/100 g of body weight.

On the first day of training, sugar pellets (Dustless Precision Sugar Pellets, ASF0042, 45 mg, Able Scientific, Australia), which serve as a reward for the 5-CSRTT, were introduced in the home cage of the animal, as a means of habituating them to the pellets. The next day (Day 1), 10 pellets were placed in the magazine of each chamber, and the animals were allowed to habituate to their respective chamber for 30 min before returning to their home cage. Animals were required to consume all sugar pellets to move on to the next task. In the first phase of training (Initial touch), any touch on the touchscreen is rewarded with a pellet, whereas in the second phase of training (Must touch), animals must touch the correct stimulus on the screen to be rewarded with a pellet, with the criteria for success outlined in Table 1. Following successful completion, animals moved on to the 5-CSRTT training phase, where the stimulus duration was gradually decreased over time.

**Table 1:** Parameters used throughout training and testing on the 5-choice serial reaction time task (5-CSRTT).

Level	Session (min)	Stim dur (s)	Time out (s)	LH (s)	ITI (s)	Criteria
Initial Touch	30	–	0	–	0	>20 Correct
Must touch	30	Unlimited	0	–	5	>30 Correct, 2 consecutive days
1	30	60	5	5	5	>80% Acc <20% Om
2	30	30	5	5	5	>80% Acc <20% Om
3	30	20	5	5	5	>80% Acc <20% Om
4	30	10	5	5	5	>80% Acc <20% Om
5	30	5	5	5	5	>80% Acc <20% Om
6	30	4	5	5	5	>80% Acc <20% Om
Post-LPS refamiliarization	30	4	5	5	5	N/A
Test day	30	2.5	5	5	5	N/A

A session begins with a sugar pellet being released. Upon retrieval, the magazine light switches off and a 5 s inter-trial interval (ITI) begins before the presentation of the stimulus light. If the animal made a response during the 5 s ITI (premature response), the house lights remained off, but no pellet will be rewarded. Following the ITI, the animal either had to nose poke the right window when the stimulus was presented or within the specified limited hold time after the stimulus light had disappeared (Table 1). If the right window was nose poked (correct response), a tone was generated, a sugar pellet was released, and the magazine light turned on. Conversely, if the wrong window was nose poked (incorrect response), the house lights turned on for 5 s (timeout) and no pellet was rewarded. If the animal did not make a response within the limited hold time (omission), there was a 5 s timeout period, with the house lights turned on and no food pellet rewarded. Following a 5 s ITI, the next trial would begin. Each training session was completed at the end of 100 trials or at 30 min, whichever came first. Animals were required to complete each stage with >80% accuracy and <20% omissions before moving on to the next stage.

Once animals had reached the criterion, they were assigned to receive either saline or LPS the following day. Animals were then allowed a rest day to recover from the effects of LPS. However, it should be noted our previous work has shown no effects on locomotion in the open field at 24 h post-dose of LPS (Corrigan et al., 2017). Following this rest day, animals were again placed in the 5-CSRTT with a 4-s stimulus duration to re-familiarise them with the task, before a probe trial was run the following day where stimulus duration was reduced to 2.5 s Response outcomes were recorded by the ABETII and extracted and saved as a Microsoft Excel file. Outcome measurements include:

• Trials completed: number of times a trial was initiated *via* nose poking the reward chamber.
• Accuracy: the number of correct responses divided by the sum of correct and incorrect responses [#correct responses/(# correct responses + #incorrect responses) × 100]. Accuracy is the main measure of attentional performance in the task.
• Percent Omissions: total number of omissions divided by the total number of trials. Percent omissions reflect the proportion of trials that end with an omission.
• Correct Response Latency: time taken to register a correct response.
• Reward Collection Latency: time taken to collect the reward.
• Premature Response Rate: total number of responses performed during the ITI (i.e., before the presentation of the light stimulus) divided by the total number of trials. Premature responses reflect response disinhibition/impulsivity in the task.
• Perseveration: total number of nose poke responses performed after either a correct or incorrect response, but before collection of the reward, divided by the total number of trials. Perseverative responses are a measure of compulsivity/cognitive inflexibility in the task.

### Serum Cytokine Determination

At 4 h post-LPS administration, a subset of animals (*n* = 5 per group) had blood collected *via* the tail vein (0.1–0.2 ml) for cytokine analysis in microfuge tubes. Samples were spun down and serum kept at −20°C until further use. A custom Milliplex mouse 4-plex cytokine kit (Millipore) was used to examine levels of IL-1β, TNFα, IL-6, and IL-10. The samples (25 μl) were loaded onto a 96 well plate in duplicate and run following the manufacturer’s instructions. Plates were read using a Magpix Luminex multiplex array (Abacus-ALS, Meadowbrook, QLD, Australia) and data expressed as pg/ml of concentration. Experimental data were calibrated against standard curves of all nine cytokines, which were fitted using a 5-parameter log fit through Analyst software (Millipore, Australia). Protein homogenates from the PFC, which had been extracted in standard RIPA buffer were also run on the Milliplex mouse 4-plex cytokine kit, but values fell below the limit of detection.

### Immunohistochemistry

Formalin-fixed brains were sucrose protected and then frozen in OCT. Twenty micrometer sections were taken from the PFC (two sections at +3.8 cm and +3.3 mm relative to Bregma). For immunohistochemistry, endogenous peroxidases were blocked with methanol/hydrogen peroxide (0.5%), followed by antigen retrieval in citrate buffer. The sections were then incubated with 30% normal horse serum for 1 h, before incubation overnight at room temperature with the IBA1 antibody (ab112, Wako: 1:1,000). The next day, the appropriate biotinylated secondary antibody (BA-9500–1.5, Vector: 1:250) was applied for 30 min, followed by streptavidin horseradish peroxidase (SA-5004–1, Vector, 1:1,000) for 60 min, with the bound antibody detected with 3,3-diaminobenzidine tetrahydrochloride (Sigma). Slides were digitally scanned using a Nanozoomer, with Z-stack acquired at 1 μm intervals. Consecutive Z-stack Images were converted to a maximum intensity projection image using ImageJ software. Using the Image5D plugin (ImageJ, NIH), z-stack images were condensed into a maximum intensity projection image. A series of 40× images were taken from the prelimbic, infralimbic, and anterior cingulate regions of the prefrontal cortex for each section (16 images in total), with results pooled across all three regions. Images were converted to binary, with surrounding processes removed manually in Fiji, thereby isolating a total of 20 complete microglia per rat, with more than one microglia isolated from some sections. We used the Sholl analysis plugin (Ferreira et al., 2014) with the first shell set at 10 μm and subsequent shells set at 5 m step sizes, to determine intersections at each Sholl radius. This also provided the critical radius (radius value with the highest number of intersections), the process maximum (the highest number of intersections regardless of radius value), the number of primary branches (intersections at the first Sholl radius), and maximum branch length (the further distance where an intersection was recorded). We also measured the soma size in Fiji and manually counted the mean number of microglia per image as expressed as cells/mm^2^.

### Data Analysis

All data were analyzed using IBM SPSS Statistics 27. There were no outliers removed for the final analysis, as no animals scored more than two standard deviations from the mean value for any measurement. For learning in the 5-CSRTT (ie number of trials to reach criterion), a two-way analysis of variance (ANOVA), with injury and LPS group allocation as the between-subjects factors, was conducted. *Post hoc* testing was conducted according to Tukey’s method.

For probe trial data, a two-way multivariate ANOVA (MANOVA) was conducted on all probe trial measures, with injury group and LPS as between groups factors. Pairwise comparisons were conducted using Bonferroni’s adjustment for multiple comparisons. Serum cytokine expression and microglial Sholl analysis were similarly analyzed by MANOVA, with injury group and LPS as between groups factors, and pairwise comparisons were conducted using Bonferroni’s adjustment for multiple comparisons. Where a significant LPS*injury interaction was found, a two-way ANOVA was conducted with *post hoc* testing conducted according to Tukey’s method. The number of IBA1+ve cells was similarly analyzed *via* a two-way ANOVA. For all testing, *p*-values <0.05 were considered statistically significant.

## Results

### Acute Response to Injury

Righting reflex time was examined *via* two-way ANOVA, with a main effect of injury (*F*_(1,47)_ = 22.59, *p* < 0.0001, 336.81 ± 136.08 vs. 188.16 ± 70.36 s, but no effect of LPS group allocation (*F*_(1,47)_ = 0.69, *p* = 0.41), nor interaction (*F*_(1,47)_ = 0.59, *p* = 0.45).

### Peripheral Inflammatory Response to LPS

**Figure 1:**
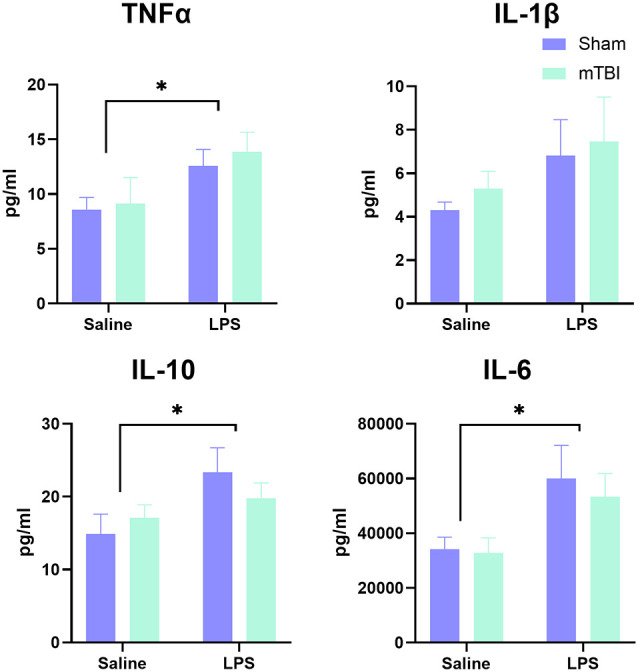
Analysis of peripheral cytokine expression at 4 h post-LPS administration in a subset of animals (*n* = 5 per group). A significant effect of LPS administration was found for IL-6, TNF, and IL-10, but not IL-1β, with no difference between injured and sham animals in response to LPS. *p < 0.05, saline vs. LPS treatment.

Serum was examined at 4 h post-LPS administration in a subset of animals to confirm induction of a peripheral inflammatory response (Figure 1). Overall, examination of cytokine levels found a significant main effect of LPS administration (Pillai’s Trace = 0.65; *F*_(4,13)_ = 5.91, *p* < 0.01), with serum levels of TNFα (13.23 ± 3.53 vs. 8.86 ± 3.90 pg/ml, *p* < 0.05), IL-6 (56,614.01 ± 22,459.13 vs. 33,465.859 ± 10,546.98 pg/ml, *p* < 0.05) and IL-10 (21.57 ± 6.20 vs. 16.00 ± 4.99 pg/ml, *p* < 0.05), but not IL-1β (7.14 ± 3.94 vs. 4.81 ± 1.40 pg/ml, *p* = 0.11), significantly increased in LPS-treated animals relative to saline. Prior injury had no effect on the peripheral response to LPS (Pillai’s Trace = 0.12; *F*_(4,13)_ = 0.43, *p* = 0.78), nor was there an LPS*injury effect (Pillai’s Trace = 0.11; *F*_(4,13)_ = 0.411, *p* = 0.80).

### Five-Serial Reaction Time Task

Performance in the 5-CSRTT was examined using a 2 × 2 multivariate analysis of variance across the various measures of performance. Animals were first required to complete 5-CSRTT training, with the final stage requiring more than 80% accuracy and less than 20% omissions with a stimulus time of 4 s. There was no difference in the number of days taken to reach criterion, with no main effect of either injury (*F*_(1,46)_ = 2.80, *p* = 0.10; sham 14 (12.75–15) vs. injury 14 (13.25–16)) or LPS treatment (*F*_(1,46)_ = 2.34, *p* = 0.13; saline 14 (12.25–15); LPS: 14.5 (13.25–16)). As such the probe trial was conducted from 35 to 45 days post-injury.

**Figure 2:**
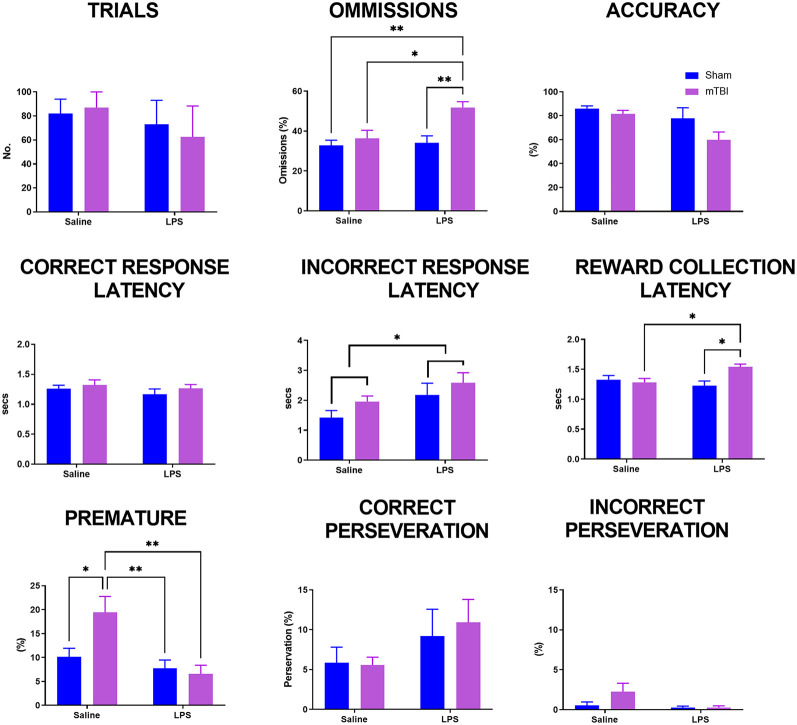
Analysis of performance on the 5-CSRTT in a probe trial where stimulus duration was reduced to 2.5 s. Previously injured animals administered LPS had an increase in both omissions and reward collection latency. Following injury, animals had an increase in premature response rate which was prevented following administration of LPS (sham:saline *n* = 14, sham:LPS *n* = 12, mTBI:saline *n* = 14; mTBI:LPS *n* = 12). *p < 0.05, **p < 0.01.

Upon reaching the criteria, animals were allocated to receive either saline or LPS the following day and then given a rest day to reduce any effects on locomotion. The 5-CSRTT was then run again at 4 s stimulus duration to consolidate previous training before animals were then challenged with a decrease in stimulus time to 2.5 s (Figure 2). A significant interaction was found for LPS*injury (Pillai’s Trace = 0.51; *F*_(9,37)_ = 2.09, *p* < 0.05) with a significant main effect of both injury (Pillai’s Trace = 0.45; *F*_(9,37)_ = 3.39, *p* < 0.01) and LPS treatment (Pillai’s Trace = 0.43; *F*_(9,37)_ = 2.09, *p* < 0.01). Injury significantly increased the number of omissions (43.90 ± 2.11 vs. 33.33 ± 2.12, *p* < 0.01), but had no significance on any other parameter examined. LPS treatment also significantly increased omissions (43.60 ± 2.27 vs. 33.63 ± 2.04%, *p* < 0.01), as well as decreasing the premature response rate (7.33 ± 1.53 vs. 13.58 ± 1.38%, *p* < 0.01) and increasing incorrect response latency (2.42 ± 0.22 vs. 1.68 ± 0.20 s, *p* < 0.05).

A significant LPS*injury interaction was found for omissions (*p* < 0.05), reward collection latency (*p* < 0.05) and premature response rate (*p* < 0.05), but not trials completed (*p* = 0.22), accuracy (*p* = 0.12), correct response latency (*p* = 0.30), incorrect response latency (*p* = 0.76) or either correct (*p* = 0.26) or incorrect perseveration (*p* = 0.89). *Post-hoc* analyses found that the number of omissions was not affected by either injury (sham:saline 32.49 ± 2.03 vs. TBI:saline 36.31 ± 3.65, *p* = 0.97) or LPS treatment alone (sham:saline 31.49 ± 2.03% vs. sham:LPS 35.46 ± 3.46%, *p* = 0.98), with only TBI:LPS animals having a significantly higher rate of omissions than all other groups (50.27 ± 3.10%, *p* < 0.01). Similarly, reward collection latency was not affected by either injury (sham:saline 1.33 ± 0.07 vs. TBI:saline 1.21 ± 0.07 s, *p* = 0.55) or LPS treatment alone (sham:saline 1.33 ± 0.07 vs. sham:LPS 1.19 ± 0.04 s, *p* = 0.38). Only TBI:LPS animals took significantly longer to collect the reward compared to TBI:saline and LPS:sham animals (1.52 ± 0.05 s, *p* < 0.01), with no significant difference compared to sham:saline animals (*p* = 0.12). In contrast, premature response rate was significantly increased in TBI:saline animals (19.07 ± 3.75%) compared to all other groups (sham:saline 5.16 ± 1.03%, *p* < 0.01; sham:LPS 7.75 ± 1.71%, *p* < 0.05; TBI:LPS 7.17 ± 1.86, *p* < 0.01).

### Analysis of Microglial Morphology Within the Pre-frontal Cortex

**Figure 3:**
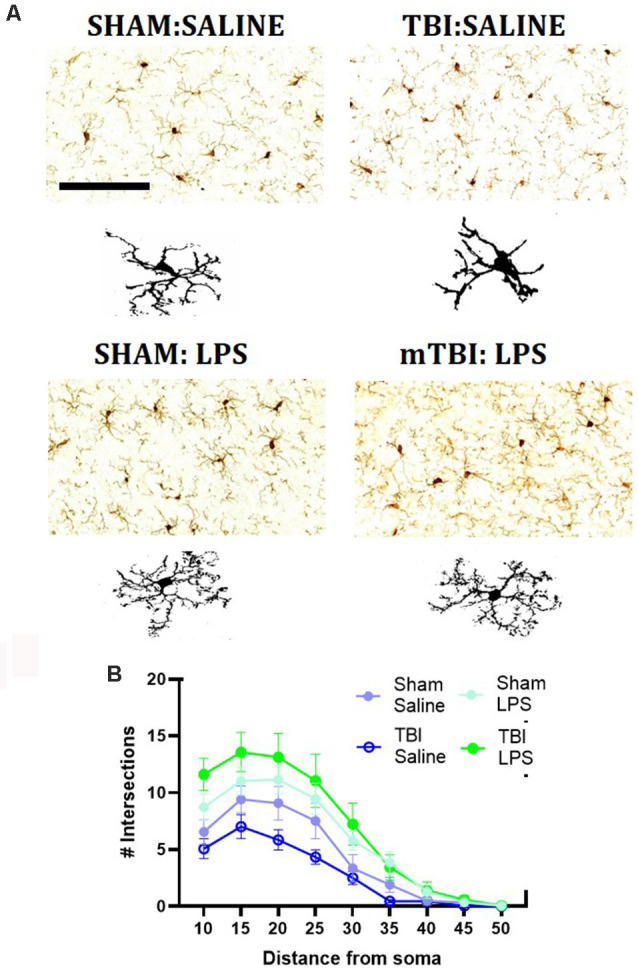
Representative images of IBA1 immunostaining within the mPFC **(A)**, with representative microglial skeletons used for analysis **(B)** and analysis of morphology *via* Sholl plot **(B)**. Scale bar = 200 μm.

We then performed immunohistochemical staining for IBA1, a marker of myeloid cells within the pre-frontal cortex (Figure 3). We quantified the number of IBA1+ve cells within the pre-frontal cortex, with a main effect of injury (*F*_(1,24)_ = 8.62, *p* < 0.01), driven by an increase in IBA1+ve cells in injured animals (63.91 ± 10.31 vs. 54.35 ± 5.63 cells/mm^2^). No main effect of LPS (*F*_(1,24)_ = 0.27, *p* = 0.61) nor injury*LPS interaction (*F*_(1,24)_ = 0.32, *p* = 0.43) was noted.

A Sholl analysis plot suggested that injury decreased the complexity of branching in microglia in TBI:saline animals, with LPS administration increasing complexity (Figure 3B). Data summarized from the Sholl analysis (Table 2) found a significant interaction between injury*LPS (Pillai’s Trace = 0.7; *F*_(6,19)_ = 8.83, *p* < 0.001), with main effects of both injury (Pillai’s Trace = 0.65; *F*_(6,19)_ = 5.96, *p* < 0.01) and LPS treatment (Pillai’s Trace = 0.84; *F*_(6,19)_ = 16.00, *p* < 0.001). A main effect of injury was only found for maximum branch length (38.21 ± 2.23 vs. 42.03 ± 4.27, *p* < 0.001). The main effect of LPS treatment was found for primary branches (9.63 ± 0.68 vs. 5.74 ± 0.68, *p* < 0.001), the critical radius (16.18 ± 0.40 vs. 12.97 ± 0.40, *p* < 0.001), total intersections (30.21 ± 15.09 vs. 54.45 ± 17.09, *p* < 0.0001), and soma area size (46.45 ± 1.31 vs. 39.93, *p* < 0.01).

**Table 2:** Sholl analysis parameters.

	Sham + saline	Sham + LPS	TBI + saline	TBI + LPS	LPS* injury	Injury	LPS
Primary branches	6.55 ± 2.67	8.66 ± 2.29	5.08 ± 2.32	10.98 ± 3.24	0.10	0.73	<0.001
Process maximum	8.39 ± 1.53	10.43 ± 4.69	5.91 ± 1.52	14.48 ± 4.09^**, #^	<0.001	0.52	<0.001
Critical radius (μm)	12.53 ± 2.68	14.88 ± 3.64^#^	12.41 ± 1.68	16.12 ± 2.09**	0.48	0.24	<0.001
Maximum branch length (μm)	42.86 ± 3.30***	41.20 ± 2.48**	36.39 ± 2.05	40.21 ± 1.80*	<0.001	<0.001	0.81
Total intersections	38.71 ± 18.76	50.77 ± 13.13*	22.94 ± 5.31	57.60 ± 20.37**	<0.05	0.46	<0.001
Soma area (μm^2^)	43.14 ± 8.93	44.03 ± 5.80	41.37 ± 9.35	44.23 ± 7.06	0.40	0.61	0.89

*n* = 7 per group, *p < 0.05, **p < 0.01, ***p < 0.001 compared to TBI:saline, ^#^compared to sham:LPS.

A LPS*injury effect was then found for process maximum (*p* < 0.05), maximum branch length (*p* < 0.001) and the total number of intersections (*p* < 0.05), but not primary branches (*p* = 0.10), critical radius (*p* = 0.48) or soma area (*p* = 0.40). *Post-hoc* analysis found that LPS administration following prior injury led to a significant increase in complexity with an increase in the process maximum (the maximum number of intersections; 5.91 ± 1.52 vs. 14.48 ± 4.09, *p* < 0.01), increase in the maximum branch length (36.39 ± 1.05 vs. 40.21 ± 1.80, *p* < 0.05) and increase in the total number of intersections (22.94 ± 5.31 vs. 57.60 ± 20.39, *p* < 0.05). This effect was not seen with LPS administration in sham animals (process maximum: 8.39 ± 1.53 vs. 10.43 ± 4.69, *p* = 0.71; maximum branch length: 42.86 ± 3.29 vs. 41.20 ± 2.48, *p* = 0.60; total intersections: 38.71 ± 18.75 vs. 38.71 ± 18.76, *p* = 0.54).

## Discussion

A prior injury in adolescence altered the response to an acute peripheral immune challenge concerning performance on the 5-CSRTT. However, this appeared to reflect a decrease in motivation, rather than attentional deficits, with a significantly higher rate of omissions and an increase in reward collection latency observed in response to LPS administration. This lack of motivation also negated the increase in premature response rate seen in injured animals. The injury did not affect the peripheral response to LPS administration with both sham and TBI animals showing a similar increase in serum cytokine levels at 4 h post-administration. However, morphological differences in microglial appearance in the mPFC were seen, with an overall effect of LPS administration on promoting hyper-ramification, although this effect was more pronounced in previously injured animals.

Previously injured animals appeared to be less motivated in the 5-CSRTT following LPS administration, with increased omissions and increased latency to collect the reward. No effect of LPS on these parameters was seen in sham animals, mirroring earlier work where a 1 mg/kg dose of LPS did not affect reward collection latency in young-middle-aged rats (Yegla and Foster, 2019). Motivated behavior depends on reward and incentive motivation, which describes the willingness to expend effort to obtain a reward (Berridge et al., 2009). A reduction in motivation is a well-described component of sickness behavior, encompassing symptoms including fatigue, depression, and apathy. The temporal profile of the development of these symptoms matches the rise of peripheral inflammatory cytokines (Bluthé et al., 2000), which induce a central inflammatory response *via* several routes including circumventricular organs, vagal afferents, and interactions with the brain endothelium (Roth et al., 2004). Central inflammation then results in this evolutionarily adaptive physiological sickness response, which typically resolves within 24 h in response to a mid-range LPS dose (0.175–0.63 μg/kg), mirroring the return to baseline of peripheral cytokine expression (Bluthé et al., 2002; Biesmans et al., 2016). However, the sickness response may be followed by a delayed period of anhedonia (2–3 days post LPS), which relates to the dose administered or the underlying inflammatory status of the brain (Godbout et al., 2005). For example, high dose LPS (830 μg/kg) reduces preference for sweetened solution up to 96 h *post-dose* (Biesmans et al., 2016), whereas lower doses (50–200 μg/kg) are associated with a much shorter period of reduced preference seen at 4 h, but not 13 h post-dose (Yirmiya, 1996). Although preference for the sweetened solution is typically thought to reflect anhedonia, the inflammatory effects on motivation appear to relate to reduced effort expenditure, rather than deficits in reward processing (Chudasama et al., 2003; Paine et al., 2011; Asinof and Paine, 2014). Effort expenditure is in part governed by the mPFC which integrates motivational, reward, and cognitive information (van Heesch et al., 2014; Yeh et al., 2015), with reduced motivation associated with mPFC dysfunction (Felger and Treadway, 2017; Michely et al., 2020). Such a process could drive the increase in omission rate noted here in the mTBI:LPS animals. Indeed, lack of motivation following repeated concussive insults in high school and college athletes is seen clinically, whereby repeated injuries are associated with higher self-reported apathy, with the threshold of injuries needed higher than that for cognitive deficits (Norris and Kipnis, 2019).

It should be noted that the increased omission rate could also reflect impairment of sustained attention, given that the rats initiated trials at the same rate as the other cohorts (Asinof and Paine, 2014). Attention is similarly mediated by the mPFC, with either over-activation (Paine et al., 2011) or excitotoxic lesions (Chudasama et al., 2003) of the mPFC associated with increased omissions in the 5-CSRTT, but these are typically associated with impaired accuracy as well, which was not found in the current study. A broader behavioral battery is required to determine the specific domains affected by a peripheral immune stimulus following mTBI, given that a decrease in motivation may mask any subtle cognitive changes. Of note, LPS administration also prevented the increase in impulsivity caused by prior injury, an effect that we have previously reported and attributed to increased dopaminergic signaling in injured animals (Kaukas et al., 2021). Administration of a single low-dose of LPS (100 μg/kg) acutely increases the enzymatic breakdown of dopamine (van Heesch et al., 2014) and decreases tissue levels of dopamine (Yeh et al., 2015). Indeed, cytokines can potentially affect multiple aspects of dopamine function, leading to decreased synthesis, impaired packaging and release, increased reuptake, or decreased dopamine receptors (Felger and Treadway, 2017), providing a potential mechanistic explanation for the ability of LPS treatment to reduce impulsivity. Reduced dopamine is also associated with lower effort-based expenditure (Michely et al., 2020) and may thus also account for the decreased motivation seen post-LPS in injured animals. Future work is needed to evaluate the specific effects of acute inflammatory insults on dopaminergic neurotransmission and whether the effects are transient or more long-lasting.

The susceptibility of mTBI animals to LPS may relate to injury-induced priming of microglia. Microglia are the innate immune cells of the CNS and responsible for interpreting and propagating inflammatory signals that affect neuronal function (Norris and Kipnis, 2019). TBI may act to prime microglia, a phenomenon whereby microglia do not display acute reactivity but become hyper-reactive after the immune stimulus. This phenomenon has been shown 1 month following moderate midline fluid percussion injury (Fenn et al., 2014; Muccigrosso et al., 2016), as well as 5 days post mTBI in adults (Collins-Praino et al., 2018). Here, we suggest that this also occurs following mTBI in mid-adolescence. The injury was found to increase the number of IBA1+ve cells within the mPFC, with Sholl analysis suggested a decrease in complexity in response to injury in this study, although the only main effect of injury was a decrease in maximum projection. This is, however, in line with previous reports on the morphological appearance of primed microglia, which have shortened and flattened cellular processes and a reduction in ramification, which would decrease the length of the maximum projection (Streit et al., 2004; Ta et al., 2019).

To confirm whether these are primed microglia, additional confirmation would be required *via* the use of alternate microglial markers (MHC-II or CD68; Fenn et al., 2014; Muccigrosso et al., 2016) or examination of the cytokines released in response to LPS. Unfortunately, here, pre-frontal cortex tissue was not examined until 4 days post-dose, and levels fell below detectable limits on the assay used.

Nonetheless, a significant LPS*injury interaction was found whereby the previous injury exacerbated microglial morphological changes in response to LPS, with an increase in process maximum, maximum branch length, and the total number of intersections. This is in line with descriptions of hyper-ramification of microglia, where microglia process length and branching are increased (Fontainhas et al., 2011). This contrasts to other reports where higher doses of LPS (1 mg/kg) have been found to induce the development of amoeboid microglia, with reduced and retracted processes (Hines et al., 2013; Savage et al., 2019), reflecting increased phagocytic capacity. Hyper-ramification of microglia within the mPFC has been seen previously following both single acute (Kongsui et al., 2015) and chronic intermittent low-dose LPS (Siemsen et al., 2020), as well as following chronic stress (Hinwood et al., 2012), ischaemic stroke (Morrison and Filosa, 2013), and TBI (Morrison et al., 2017). The functional significance of this phenotype is not yet clear, with hyper-ramified microglia in response to stress not thought to release cytokines, but rather β1-integrin, in response to the disruption of the extracellular matrix (Hinwood et al., 2012). Indeed it is thought these microglia may be involved in neuronal circuit rewiring by increasing the frequency and density of potential contacts with neurons in each microglial cell’s “territory” (Wu et al., 2015). The microglial phenotype may also change in response to alteration in neurotransmission, with glutamatergic neurotransmission shown to induce hyper-ramification (Fontainhas et al., 2011), with increased process number and process extension (Eyo et al., 2014). This is in part driven by the expression of glutamatergic receptors on microglia, with inflammation known to increase glutamatergic receptors, like mGlu-5 on microglia (Drouin-Ouellet et al., 2011) and LPS to increase glutamatergic signaling (Chan et al., 2020). Exposure to a prior inflammatory stimulus, in mTBI (Donat et al., 2017), could alter the microglial expression of these receptors and hence increase the response of microglia to later LPS. Indeed, hyper-ramification of microglia was seen in response to the combination of two stressors (maternal separation + food deprivation in adulthood), but not either alone, suggesting it may relate to exposure to repeated inflammatory events (Drouin-Ouellet et al., 2011). Further research is needed to evaluate the functional significance of hyper-ramification in microglia and how it may relate to low-grade repeated inflammatory stimuli, like those utilized here.

It should be noted that these results occurred on a background of chronic mild food restriction to facilitate performance on the 5-CSRTT, with food restriction known to have potential anti-inflammatory effects (Matsuzaki et al., 2001; Horrillo et al., 2011). However, this may be age-dependent, with Yegla and colleague’s finding that only middle-aged (12 months), but not young (4 months old), animals showed a reduction in peripheral cytokine expression and microglial activation in response to LPS on a background of food restriction (Yegla and Foster, 2019). This may relate to continued weight gain in laboratory rats over the lifespan, driving the release of more adipose tissue-derived pro-inflammatory factors (Shin et al., 2015). Thus, given the age of the animals utilized here (11–12 weeks), the anti-inflammatory effects of food restriction should have been limited.

In summary, a prior mTBI during adolescence subtlety alters the central response to remote systemic LPS administration in adulthood, appearing to prolong the sickness response with deficits noted in motivation on the 5-CSRTT and morphological changes suggestive of hyper-ramification in the mPFC. This supports the idea that mTBI is a chronic event and alters susceptibility to later acute inflammatory stimuli, which should be taken into account during recovery.

## Data Availability Statement

The raw data supporting the conclusions of this article will be made available by the authors, without undue reservation.

## Ethics Statement

The animal study was reviewed and approved by University of Adelaide Animal Ethics Committee.

## Author Contributions

LK, JK and FC carried out the experiment. FC wrote the manuscript with support from LK, JK and LC-P. LC-P and FC helped supervise the project, and design the original experiment. All authors contributed to the article and approved the submitted version.

## Conflict of Interest

The authors declare that the research was conducted in the absence of any commercial or financial relationships that could be construed as a potential conflict of interest.
